# P-212. Outcomes Associated with Bezlotoxumab Use

**DOI:** 10.1093/ofid/ofae631.416

**Published:** 2025-01-29

**Authors:** Amy Kole, Daniel Schreck, Robert Orenstein, Robert Kirchoff

**Affiliations:** Mayo Clinic Arizona, Scottsdale, Arizona; Mayo Clinic Arizona, Scottsdale, Arizona; Mayo Clinic in Arizona, Phoenix, AZ; Mayo Clinic Arizona, Scottsdale, Arizona

## Abstract

**Background:**

Bezlotoxumab is a monoclonal antibody infusion approved to prevent recurrence of *Clostridioides difficile* infection (CDI). The objective of this study was to explore outcomes associated with bezlotoxumab use at three academic medical centers.

**Figure:**
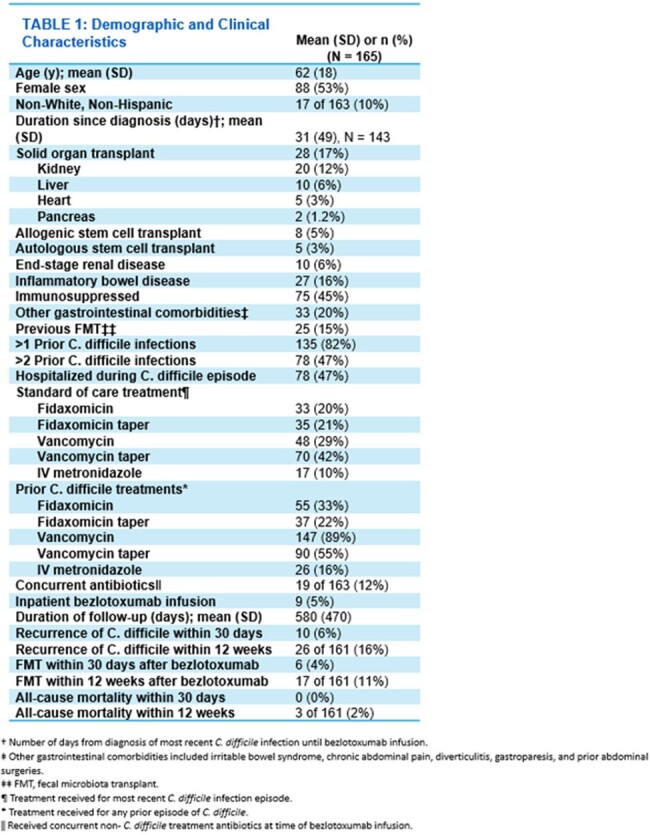

**Methods:**

A retrospective cohort study was conducted with 180 patients who received bezlotoxumab from May 2018 through November 2023. The primary outcome was recurrence of CDI within 12 weeks of receipt of bezlotoxumab. The study also sought to evaluate risk factors for CDI recurrence in patients who received bezlotoxumab.

**Figure:**
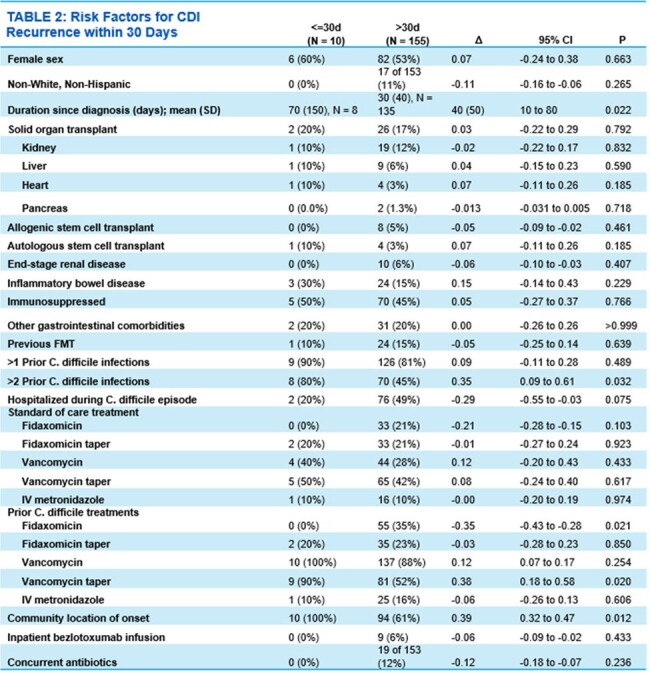

**Results:**

Of the 180 patients who received bezlotoxumab at our institution, 165 were included in the study. Fifteen patients were excluded because they did not improve with standard of care CDI treatment. In the study cohort, 45% were immunosuppressed (n=75), 17% had a solid organ transplant (n=28), and 16% had a history of inflammatory bowel disease (n=27). Ninety-five percent of patients received bezlotoxumab as an outpatient (n=156). Recurrence of CDI within 12 weeks occurred in 16% of patients. Duration from diagnosis of CDI to bezlotoxumab infusion (p=0.022) and having greater than two prior CDI episodes (p=0.032) were statistically significant predictors for CDI recurrence within 30 days.

**Figure:**
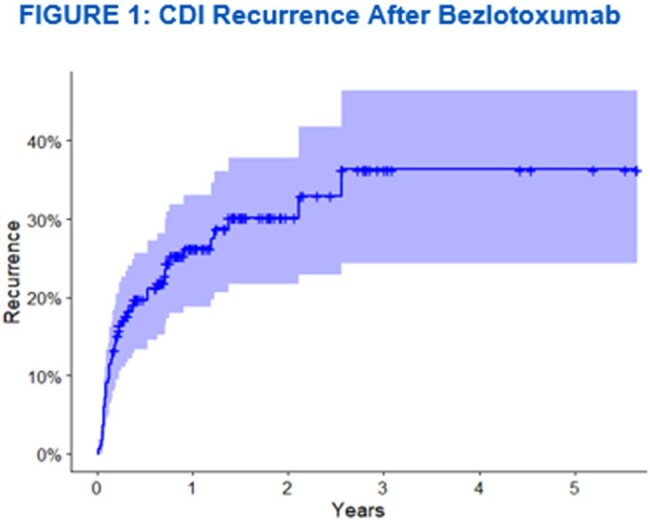

**Conclusion:**

Our study demonstrated similar CDI recurrence rates for patients who received bezlotoxumab in a real-world setting compared to previous clinical trials. Patients with CDI may benefit from receiving bezlotoxumab earlier in the course of illness and prior to developing multiple recurrences.

**Disclosures:**

**Robert Orenstein, DO, FACP**, Ferring: Advisor/Consultant|Ferring: Honoraria

